# Altering physiological networks using drugs: steps towards personalized physiology

**DOI:** 10.1186/1755-8794-6-S2-S7

**Published:** 2013-05-07

**Authors:** Adam D Grossman, Mitchell J Cohen, Geoffrey T Manley, Atul J Butte

**Affiliations:** 1 Department of Bioengineering, Stanford University, Stanford, CA, USA. Now at Praedicat, Inc., Culver City, CA, USA; 2 Department of Surgery, University of California San Francisco, San Francisco, CA, USA; 3 Department of Neurosurgery, University of California San Francisco, San Francisco, CA, USA; 4 Department of Pediatrics and the Department of Medicine, Stanford University School of Medicine, Stanford, CA, and Lucile Packard Children's Hospital, Palo Alto, CA, USA

## Abstract

**Background:**

The rise of personalized medicine has reminded us that each patient must be treated as an individual. One factor in making treatment decisions is the physiological state of each patient, but definitions of relevant states and methods to visualize state-related physiologic changes are scarce. We constructed correlation networks from physiologic data to demonstrate changes associated with pressor use in the intensive care unit.

**Methods:**

We collected 29 physiological variables at one-minute intervals from nineteen trauma patients in the intensive care unit of an academic hospital and grouped each minute of data as receiving or not receiving pressors. For each group we constructed Spearman correlation networks of pairs of physiologic variables. To visualize drug-associated changes we split the networks into three components: an unchanging network, a network of connections with changing correlation sign, and a network of connections only present in one group.

**Results:**

Out of a possible 406 connections between the 29 physiological measures, 64, 39, and 48 were present in each of the three component networks. The static network confirms expected physiological relationships while the network of associations with changed correlation sign suggests putative changes due to the drugs. The network of associations present only with pressors suggests new relationships that could be worthy of study.

**Conclusions:**

We demonstrated that visualizing physiological relationships using correlation networks provides insight into underlying physiologic states while also showing that many of these relationships change when the state is defined by the presence of drugs. This method applied to targeted experiments could change the way critical care patients are monitored and treated.

## Background

A major goal of medical physiology has long been to fully specify a model for how molecules and systems in the body interact and react to changes in environment. Thus far the most widely known and complete model of this sort was produced by Guyton [1], systematically and mathematically detailing 354 independent elements that together regulate cardiac output. A major and necessary assumption inherent to this work and those that followed is the capability of a single set of equations - a "physiology" - to describe the ways in which systems and molecules interact. While the concept of a single unified set of relationships to define physiology has certainly served both science and medicine extremely well at the population level, we now know (and, some would argue, have always known) this view to be incorrect on the level of individuals [2]. Regardless, the literature is full of population-based studies that associate single physiological measures with outcome. For example, many links have been made between the variability of such measures - mostly heart rate variability - to various adverse outcomes in a population of trauma patients [3-7]. While valuable in advancing medical knowledge the limits of population-based medicine are becoming clearer. The rise of personalized medicine via genomics, personalized dosing algorithms [8], and genome wide association studies showing individual risk of disease are two obvious examples of such limitations of population-based medicine [9,10].

Applying the concept of patient specificity to physiology using multivariate data analysis and visualization tools has recently found some traction. Nelson *et al. *[11], using cerebral microdialysis, showed that patients exhibited individualistic physiological patterns that were also reflective of shock, but they could not find common patterns between patients. Rixen et al [12] devised a physiological state classifier based on seventeen variables collected once daily, enabling prediction of patients' deaths. Peleg et al [13] devised a biological simulator based on multivariate physiological measurements. Additionally, several patient-specific monitoring and learning algorithms have been developed to improve detection of severe and acute pathologies [14] and as triage assistance and clinical decision support systems [15,16]. Our own previous work has shown the dependence of clinical assessment on data collection frequency [17] and the benefits of combining multivariate physiological data with continuous monitoring for managing secondary brain injury and gaining clinical insight not possible without such methods [18]. We have also shown that physiological state as defined by agglomerative hierarchical clustering leads to identification of complex metabolic patterns naturally sorted for outcome measures including death and multiple organ failure [19].

One way to visualize and examine the interrelationships of physiological measurements and how they change is by using networks; this approach has become the basis for large efforts to understand the relationships between genetics and disease [20]. Networks constructed using correlated pairs of variables have found uses in many areas, including integrative biology [21], metabolic networks [22], gene expression networks [23], and even the structure of the Internet [24]. Furthermore, we have shown previously that physiological correlation networks changed based on whether patients contracted an infection during their stays in the intensive care unit (ICU) [25]. Because physiology depends on disease state, we hypothesized that the effects of another common modifier of physiology, drugs, would be apparent when visualizing physiological correlation networks and comparing the cases of the drugs being given or not. Even if drugs are used that primarily affect a single molecule, many drugs potentially have a large set of off-target or side effects making broader changes in physiology that we believe could be visualized using clinical physiological networks.

To demonstrate this we examined the effects of administering pressors to patients in the ICU by collecting high-frequency clinical physiological measurements and using them to create correlation networks during times when pressors were given during the normal course of treatment and separately for times when they were not. We then show that the structure of these networks changed when pressors were administered, with changes falling into two categories: the sign of the correlation changed and correlations that appeared or disappeared when pressors were given. We also provide an example demonstrating that both patient individuality and drug differences can visibly affect physiological relationships. These methods could open the door to improved treatment strategies for critical care patients, and may even lead to clinically-relevant models of personalized physiology gleaned from the data that are normally collected in every ICU.

## Methods

### Physiological data collection

This study was approved by and conducted under supervision of the Committee on Human Research at the University of California San Francisco. We collected physiological data from nineteen severely injured poly-trauma patients from the intensive care unit at San Francisco General Hospital between May 2004 and June 2005, as previously described [19,25]. Briefly, patients were included based on admission to the ICU and a requirement for ongoing resuscitation.

Patients were placed on respiratory support and standard ICU monitoring protocols using the bedside monitor. Nine types of physiological data (Table 1) were collected from these monitors at one-minute intervals and stored in a database using a multimodal bioinformatics system (Aristein Bioinformatics, Palo Alto, CA) that integrates continuous data from the ventilator with other continuous and intermittent measurements for a total of twenty-nine physiological data streams. Clinical blood gas sampling was supplemented with a point-of-care analyzer (Opti CCA, Roche, Mannheim, Germany) and plasma lactate levels were measured (Accutrend^®^, Roche). Patients' non-injured deltoid muscles were fitted with a muscle oxygen catheter (Integra Neurosciences, Plainsboro, NY) as in [26] to measure partial pressure of oxygen in muscle. Catheters and monitoring took place for seven days or until the patient was extubated. Patients were followed until discharge or death and all complications were documented in the database.

**Table 1:** Types of data collected and their abbreviations.

Symbol	Definition
PaO_2_/PCO_2_	Arterial O_2_/CO_2 _partial pressure
MAP	Mean arterial blood pressure
HR	Heart rate
MTemp	Muscle oxygen temperature
PmO_2_	Muscle oxygen tension
SpO_2_	Oxygen saturation percentage
FiO_2_	Fraction of inspired oxygen
Gluc.L	Serum glucose
pH	Blood pH
PF	PaO_2_/FiO_2 _ratio
BE	Base excess
mLact	Muscle lactate concentration
mGluc	Muscle glucose concentration
mGlut	Muscle glutamate concentration
mPyr	Muscle pyruvate concentration
mLP	Muscle lactate/pyruvate ratio
Lact.L	Serum lactate
Gluc.F	Bedside finger-stick glucose reading
Comp	Mechanical lung compliance
PEEP	Positive end expiratory pressure
MinVol	Volume of air per minute
Ctemp	Core temperature
CVP	Central venous pressure
Hb/HCT	Hemoglobin/hematocrit
Cl^-^	Serum chloride
BUN	Blood urea nitrogen
Cr	Serum creatinine

Additional data preparation has been described elsewhere [25]. Briefly, data were collected, de-identified, integrated into a single database, and hand-curated. Muscle microdialysis data were interpolated, while all other measures were retained unaltered. All collected variables and their abbreviations are summarized in Table 1.

### Univariate data analysis

We grouped our minute-by-minute data from all patients according to whether pressors were being given and used Wilcoxon-Mann-Whitney (Mann-Whitney U) tests to determine whether the univariate physiological measures came from the same underlying population in those two cases. Bonferroni correction was used to correct for multiple comparisons with significance set at p < 0.05.

### Correlation network construction

We grouped all of our minute-by-minute data, regardless of patient, according to whether pressors were being administered during that minute, and calculated the Spearman correlation coefficient for each possible pairing of physiological measurements. We chose rank instead of linear correlation to directly show the monotonic relationships between variables without the need for them to be linear. To maximize data utility, we calculated the correlation for each pair of measurements using all those time points containing data for both variables of that pair. While this can yield a correlation matrix that is not positive definite, we are concerned with the coefficients themselves rather than manipulating the resulting matrix. We calculated the p-value for each correlation using Student's t-test on the standard z-transform and used Bonferroni correction for multiple comparisons. We considered a connection (edge) between two physiological measures (nodes) to exist if the correlation between them was statistically significant.

To assist in comparing the physiological correlation network when pressors were given ("pressors on") to the one generated when they were not ("pressors off"), we visualized the union of these two networks into three interesting and distinct cases. The first, "static", network contains those connections present in both the "pressors on" and "pressors off" networks, and with the same sign of directionality in both cases. The second, "either-or", network consists of connections present in either of the two networks, but not in both. The third, "sign-change", network consists of connections present both with and without pressors being given, but with a different sign of directionality in the two cases (i.e. positive correlation in one network and negative correlation in the other). Rather than simply display these networks as tables of correlation values we visualize them as a network to lend clarity. Each physiological variable becomes a node and each significant correlation becomes a line connecting the two relevant nodes. This way the differences in correlation direction and strength can easily be seen across the different conditions.

### Individual patient comparison

We tested the null hypothesis that the slopes of two linear regressions are equal using the test [27]:

(1) $$t=\frac{\beta_{1}-\beta_{2}}{\sqrt{\frac{\sigma_{Y_{1}}^{2}\left(1-r_{1}^{2}\right)}{\sigma_{X_{1}}^{2}\left(N_{1}-2\right)}+\frac{\sigma_{Y_{2}}^{2}\left(1-r_{2}^{2}\right)}{\sigma_{X_{2}}^{2}\left(N_{2}-2\right)}}},$$

where *β_i _*is the slope of the linear regression of the sample Y_i _on X_i_, *r_i_*^2 ^is the Pearson correlation coefficient associated with that same regression, and *σ_Xi _*indicates the sample standard deviation of *X_i_*. The resulting statistic follows a t distribution with N_1 _+ N_2 _- 4 degrees of freedom, where N_1 _and N_2 _are the numbers of samples in the first and second groups, respectively. We compared the regression slopes across the cases of receiving and not receiving pressors within two individual patients. We also compared the regression slopes of two individuals at times when they were being given pressors to times they were not.

## Results

### Patient demographics

We collected 29 different measures of clinical physiological data from 19 individuals at one minute intervals, each of which was admitted to the ICU as severely injured trauma patients. As summarized in Table 2, our patients had a mean injury severity score of 28 ± 10, were in the ICU for an average of 24 days during a mean 40 day hospital stay. Standard monitoring was initiated upon admission to the ICU while the full experimental protocol was initiated shortly thereafter. Data were then collected for a mean of 67 ± 48 hours. Each patient had a minimum of 24 hours worth of data collected, while 10 of 19 patients had at least 72 hours worth of data. The mortality rate was 16%. None of the above was associated with any treatment or outcome measure.

**Table 2:** Patient demographics.

	Frequency	Percent
**Gender:**		

Female	4	21
Male	15	79

**Outcome:**		

Live	16	84
Die	3	16

**Mechanisms:**		

Gun shot wound	9	47
Pedestrians vs. auto	3	15
Fall/Jump	2	11
Other penetrating	2	11
MV/MC crash	2	11
Bike crash	1	5

**Complications:**		

MOF	8	42
Infections	11	58

	**Mean ± s.d**.	**Range**

Age (years)	38 ± 18	18 - 72
ICU length of stay (days)	24 ± 21	1 - 78
Hospital length of stay (days)	40 ± 42	1 - 172
ISS	28 ± 10	16 - 50

**Injury sites (maximum AIS)**	**# of patients**	**% patients**

Abdomen	4	21.0
Extremity/pelvis	1	5.3
Thorax	1	5.3
Multiple	13	68.4

**Figure 1:**
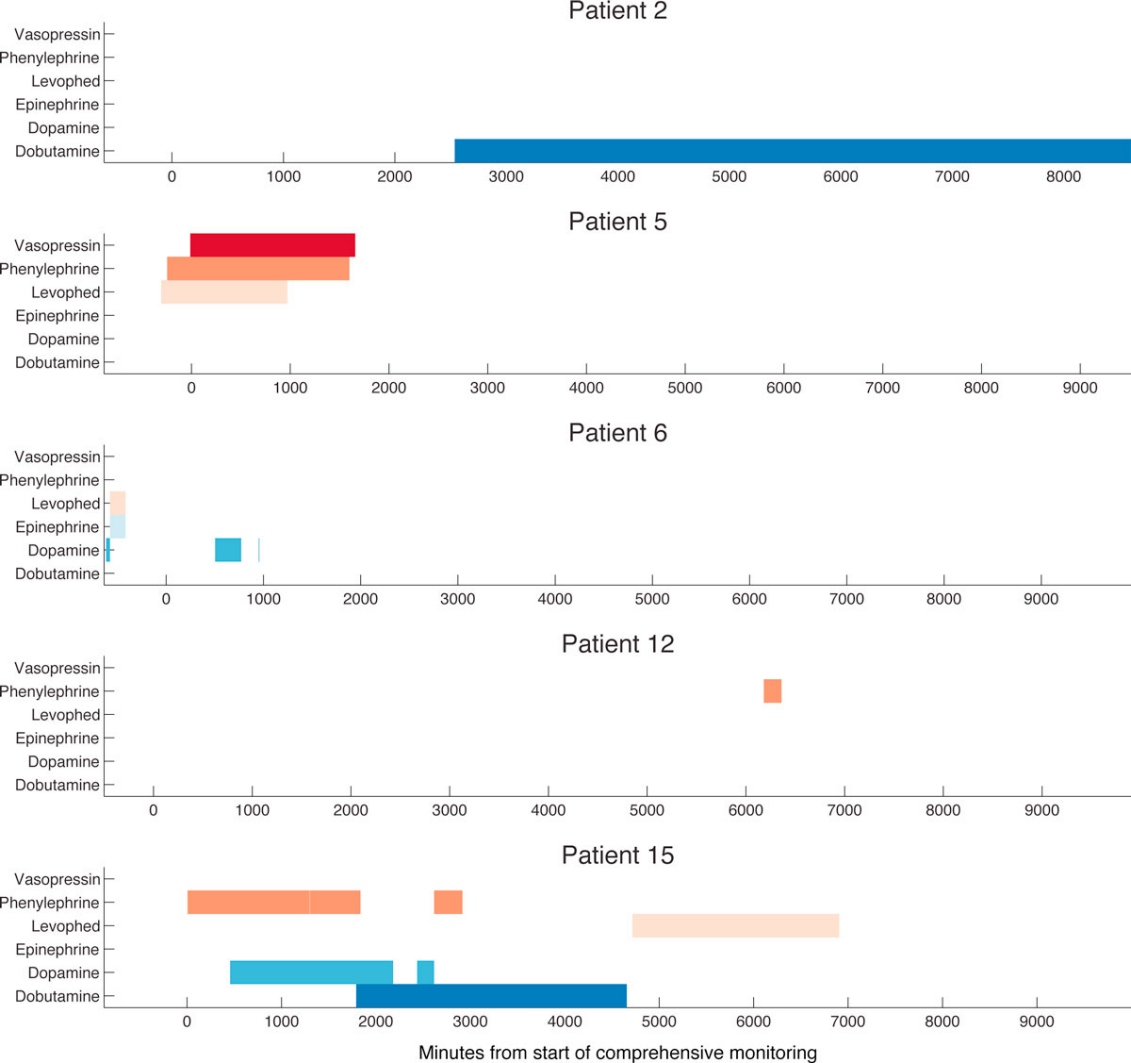
**Timing and drug specification for the five patients who received pressors**. The x-axis shows the entire time (in minutes) for which we obtained comprehensive physiological data with time zero being the start of microdialysis catheter placement.

Five of the patients received intravenous doses of pressor drugs during their stay (Figure 1). Patient 2 received solely dobutamine for the last 2/3 of the monitoring period while Patient 12 received phenylephrine for a short period in the middle of his stay. The other three patients received combinations of pressor drugs at various times during their stays; Patient 5 received simultaneous doses of three drugs for the first part of his stay. In sum, 14,428 samples (15.7%) were taken while pressors were administered out of a total of 92,000 samples across all patients.

### Effect of pressors on individual measures

**Figure 2:**
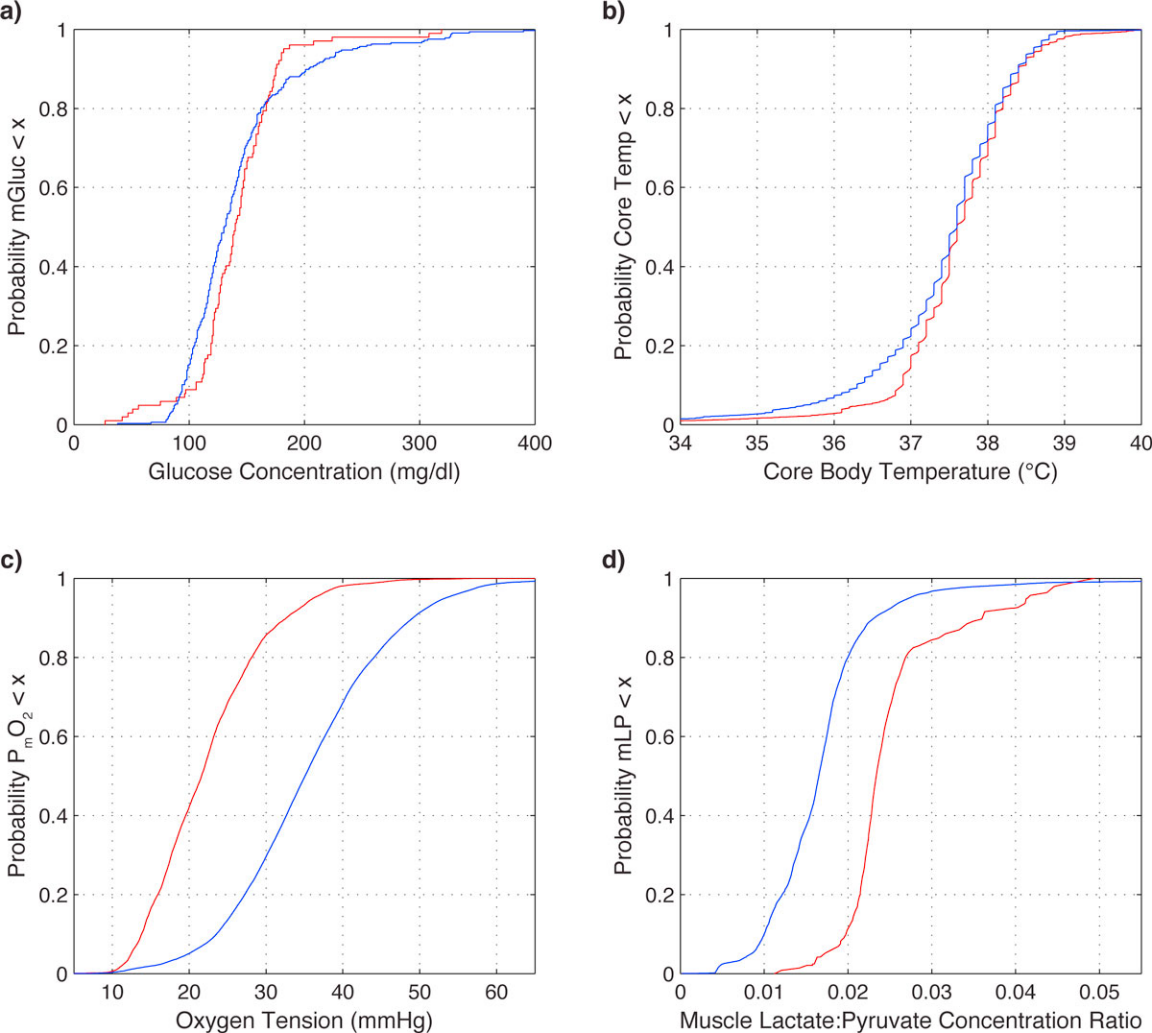
**Empirical cumulative distribution functions**. Empirical cumulative distribution functions for four physiological variables grouped according to whether pressors were being administered (red) or not (blue). (a) Finger stick glucose readings do not differ significantly. (b) Core body temperature undergoes a statistically significant difference but is unlikely to be clinically significant. (c) Muscle oxygen tension and (d) muscle lactate:pyruvate ratio both show statistically significant changes upon administration of pressors and the changes appear to be clinically different as well.

Out of 29 types of physiological measures, 22 were statistically significantly different when pressors were given. Figure 2 shows the univariate effects of administering pressor drugs on four of these physiological measures with three different types of significance. Finger-stick glucose readings do not show a statistically significant difference with or without pressors. Differences in core temperature measurements (Figure 2b) are highly significantly associated with pressor use (p = 2 × 10^-39^), but because the actual temperature shift is very small it is likely not *clinically *meaningful except for the hypothermic data points. Two other measures showed a more significant shift, both statistically and clinically. Muscle oxygen tension(Figure 2c) shows a left shift, denoting a decrease in overall muscle oxygenation in patients at the time they are receiving pressors. The lactate:pyruvate ratio for muscle undergoes a rightward shift (Figure 2d) while pressors are being administered. Cumulative distribution functions for the other variables are shown in Additional file 1. This method of univariate analysis cannot be used to ascertain cause and effect, as clinical signs of decreased muscle oxygenation likely leads clinicians to start pressors.

### The core physiological network response to pressors

We next looked at the 406 possible pairings of the 29 physiological variables. We comprehensively computed Spearman correlation coefficients for each pair of variables, using all data points when both variables were simultaneously measured. We computed pair-wise correlations separately using those data points associated with both the absence and presence of pressors. To generate a more complete picture of physiological changes concurrent with administering pressors, we used a correlation network-based analysis to study the interrelationships between our 29 clinical measurements. We visualized the differences in these correlational networks in three ways to highlight the similarities and differences in the physiological relationships when pressors were given. The "static" network is composed of those relationships that exist and have the same sign of directionality in both conditions and contains 64 out of a possible 406 edges (Additional file 2). These relationships should therefore include known physiology and serve as useful controls. For example, hemoglobin and hematocrit are strongly positively correlated both with and without pressors (ρ = 0.98, p < 10^-20 ^in both cases). The three independent glucose measurements are also all positively correlated (five of six ρ > 0.62 with p < 10^-8^, exception: muscle and lab glucose correlation was not significant with pressors on due to lack of sufficient concurrent samples), as is base excess with pH (ρ = 0.66 and 0.89, both p < 10^-16^). Muscle oxygen tension is negatively correlated with muscle lactate concentration (ρ = -0.28 and -0.65, p < 10^-20^) and the lactate:pyruvate ratio (mLP) (ρ = -0.17 and -0.25, p < 10^-20^) both with and without pressors, as would be expected since a reduction in oxygen available to muscle should lead to an increase the relative amount of anaerobic respiration and the resulting lactate. Ventilation parameters are also correlated as expected; arterial oxygen, oxygen saturation, and arterial oxygen:inspired oxygen (PF) ratio are all positively correlated, while PF ratio is anti-correlated with inspired oxygen fraction (all |ρ| > 0.46, all p < 10^-7 ^with one p = 10^-4^). These findings reassured us that our methodology was specific enough to re-establish known, and intuitive physiological relations not associated with pressors.

The second stage of analysis, the "either-or" network, (Additional file 3) contains the 48 edges that exist either while pressors were given or not, but not in both cases. Twenty-nine edges are present only when pressors are not. For example, blood urea nitrogen (BUN) and creatinine concentrations are strongly correlated without pressors (ρ = 0.60, p << 10^-60^) but with pressors have only a statistically insignificant negative correlation (ρ = -0.06, p = 1). Nineteen edges are present only when pressors are being given. As we have fewer measurements with pressors than without, these correlations may be more likely be indicative of physiological changes rather than being anomalous due to large numbers of data points. Interpreting these emerging relationships can be difficult, as some may be reflective of the reason for giving pressors while others could be a consequence of pressors. For example, the coupling between arterial and muscle oxygen is associated with pressors (ρ = 0.55, p = 10^-4^). Since under normal conditions arterial oxygen remains nearly saturated, one could hypothesize (and eventually test) that oxygen transport is impaired at times when pressors are given. While receiving pressors pH becomes strongly negatively correlated with muscle lactate (ρ = -0.76, p = 8 × 10^-8^) and with muscle pyruvate (ρ = -0.86, p = 2 × 10^-11^), possibly indicating an increased susceptibility to acidosis. Perhaps the most interesting of our three analysis cases is what we term the "sign change" network, consisting of those edges that are oppositely correlated depending on whether pressors are being administered. Thirty-nine edges fall into this case (Additional file 4). While pressors were being given, the relationships between mean arterial pressure (MAP) and 8 other measures significantly change direction, in stark contrast to the edges connecting MAP to three other measures in both the static and either-or networks. The relationships between oxygen saturation (SpO_2_) and nearly all of the muscle microdialysis measures changed from positive to negative correlations while pressors were being given.

### Individual patients

**Figure 3:**
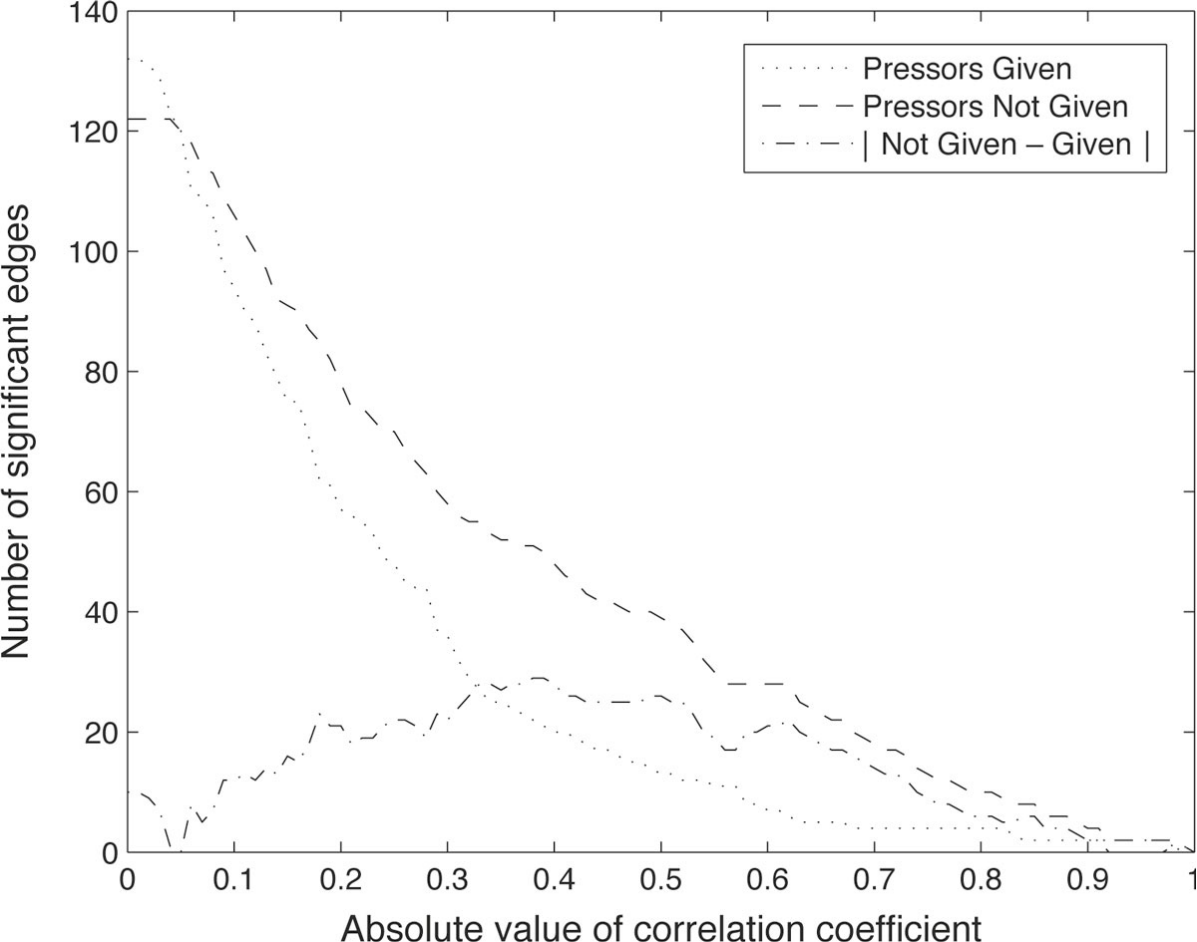
**Number of edges in the correlation networks as a function of cutoff threshold**. The third line shows the difference in the number of edges between the two conditions.

**Figure 4:**
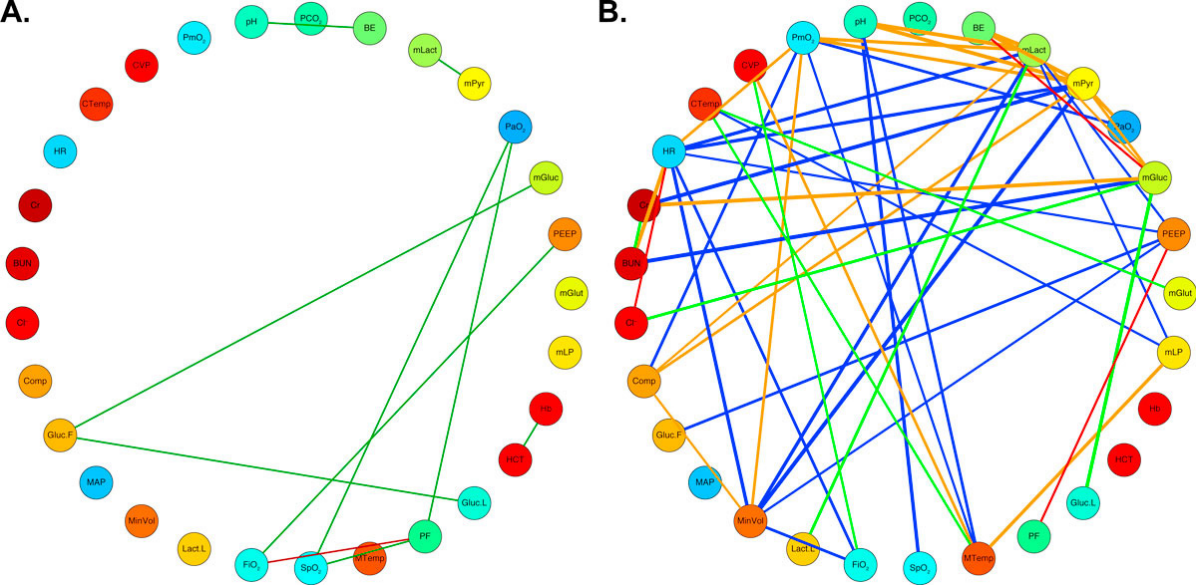
**Correlation networks**. Correlation network diagrams showing the Spearman correlation networks after applying a threshold of |ρ| > 0.4 as discussed in the main text. (a) The "static" network of edges with the same directionality both with and without pressors. Red edges indicate a negative correlation while green edges indicate a positive correlation. (b) The "either-or" network with edges present either with pressors or without, but not both. Red/green edges as in (a) indicate edges without pressors. Orange/blue edges indicate negative/positive correlations with pressors. Wider lines indicate a stronger correlation.

Having shown that drugs alter correlation networks constructed from clinical physiological measurements, we sought to determine how these changes affect individual patients. To accomplish this we sought a way to narrow our attention to a subset of the 151 edges from the three networks discussed above and consequently chose to restrict our attention to those edges that were most highly correlated in our entire patient population and defined a threshold for ρ accordingly. A cutoff of |ρ| > 0.4 provided a reasonable network size at a point where the number of connected edges remained stable with small variations in the cutoff (Figure 3). This reduced the number of edges across all three networks to 58, with a notably empty "sign change" network (Figure 4).

**Figure 5:**
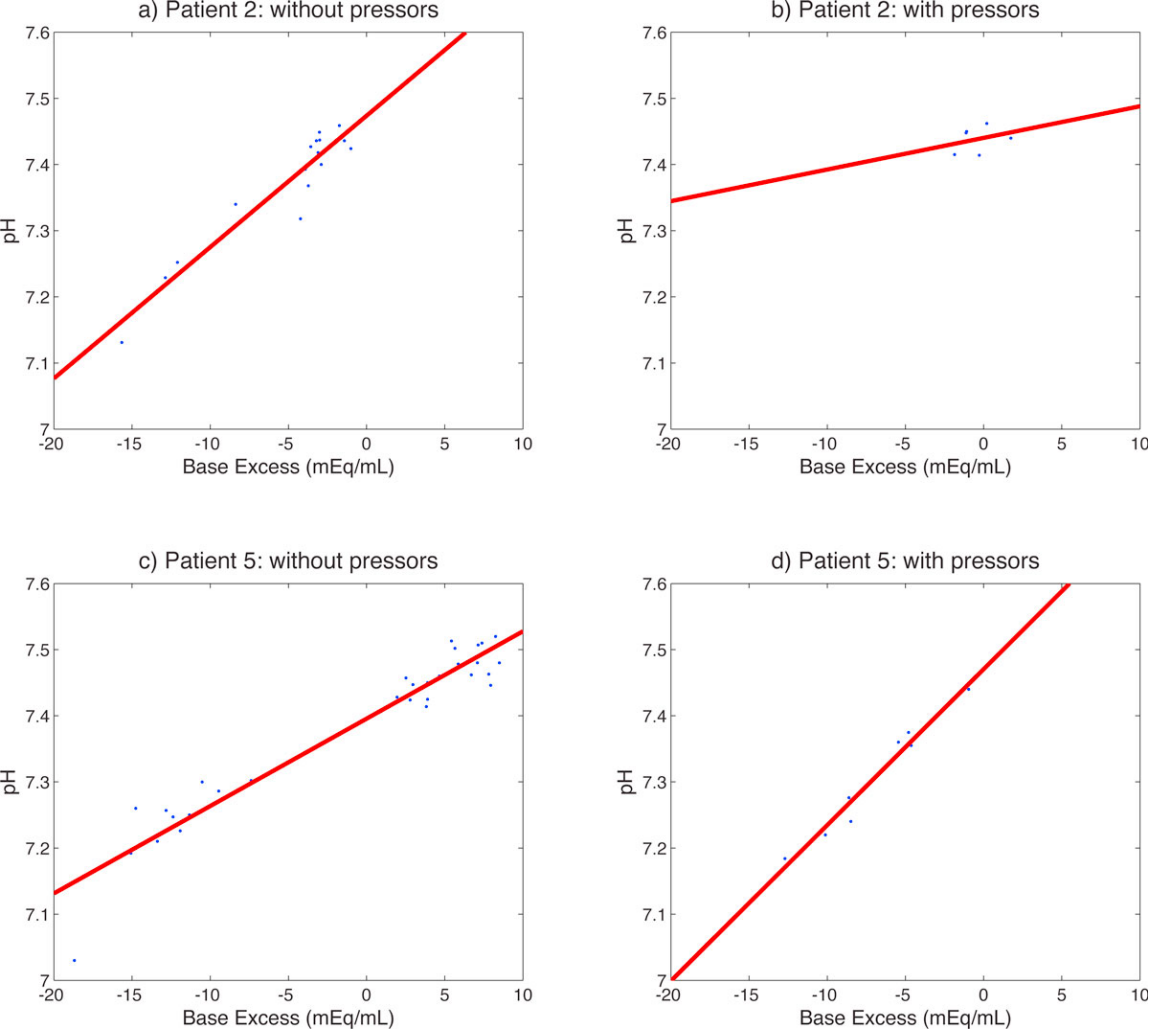
**Individual patient analysis**. Scatter plots with regression lines for base excess vs. pH in Patient 2 (a-b) and Patient 5 (c-d). Panels a and c show times without pressors while panel b and d show times with pressors.

We then singled out patients 2 and 5 to help develop a more nuanced understanding of how the global network changes manifest in these individuals. For each of the 58 relationships identified above (Figure 4) we calculated the linear regression coefficients separately when the patient was given and not given pressors and tested for significant differences in three comparisons: with and without pressors for each of patients 2 and 5, and without pressors between patients 2 and 5. This approach allows us to look not only at the strength of association between two variables, but also shows the more subtle ways in which these relationships vary. One interesting example is the relationship between base excess (BE) and blood pH (Figure 5), taken from the static network. When pressors were not being given, i.e. in the baseline state for each of patients 2 and 5, the slope of the BE/pH relationship was significantly different. When Patient 5 received pressors the slope of the relationship changed from his baseline, in contrast to Patient 2 for whom it did not. The range of both pH and BE values decreased for Patient 2 when pressors were administered.

## Discussion

We set out to show that giving drugs to ICU patients can modify their physiology on a wider scale than expected from the main effect of the drugs, and that correlation networks can be used to visualize these changes. We have shown that changes in both univariate measures and correlation networks describing physiological states are associated with pressors being given to critical care patients and demonstrated a method to visualize these differences as three distinct cases. The "static" case includes a core physiology that is not associated with pressor use, while the "sign change" and "either-or" networks show two different types of physiological change associated with pressors. After narrowing our attention to only the strongest correlations from the original correlation networks, we examined a set of 58 edges to demonstrate the subtlety with which these changes can manifest in individual patients, even in a relationship that is part of the "static" network.

Defining physiological state is a difficult yet important endeavor that could change the way critical care patients receive treatment. In previous studies by our group and others [2,12,18,19] the definition of physiologic state was based on instantaneous values or time histories of single physiological measurements. This approach has been successful, giving rise to scoring systems such as APACHE-III, the Injury Severity Score, and many systems for outcome prediction [12,16,28,29]. Those studies mainly focused either on algorithm development or outcome scores. Here, we focused more on developing a technique for a deeper understanding of physiology, using a novel application of building correlation networks from temporally dense data.

The power of network-based analysis to discover relationships and improve clinical insight is gaining prominence [20]. Constructing correlation networks out of readily measured clinical physiological variables can be the first step towards moving these innovative research tools to the clinic. One primary use for these networks might be in customization of drug therapies. For example, Adourian, et al., have used correlation networks to identify biomarkers for hepatic drug toxicity [21], but also make the point that empirical approaches using easily acquired data are likely to shed more light on complex physiology than normal univariate procedures.

One potential criticism of this work is that the use of a correlation network analysis on metabolites and variables yields results that are already known and generally well understood. However, it has been shown that similarly constructed correlation networks do not match up with those expected from interaction networks and that both approaches contribute useful information [22]. Our work provides more evidence in favor of visualizing physiology via correlation networks, as ours contain unexpected associations that do not conform to standard physiological thinking. Establishing causation is a difficulty in this work, as in all correlation network analyses. In this case, some of the correlations associated only with pressors are likely to be reflective of the reasons for giving the drugs while others are likely to be due to the drugs. It is likely, for example, that the negative correlation that appears between muscle oxygen tension and heart rate when pressors are given reflects impairment in peripheral oxygen transport that drives the cardiovascular system to work harder [30], resulting in increased heart rate that is among the diagnostic criteria for shock [31]. At the same time, the uncoupling of BUN from creatinine during pressor administration while associating with heart rate and muscle glucose does not have any clear connection to shock or pressors. Notwithstanding this inherent ambiguity, our method is capable of suggesting putative physiological relationships associated with the physiologic state of a patient that could shed light on poorly understood aspects of physiology. Future work can look at how relationships between measurements change over time, and can provide temporal evidence for causation by examining network changes immediately preceding and following the start of pressors.

To draw clinically useful conclusions about this "new" physiology, one must also improve on our main limitation of having 19 patients receive five different pressors in various combinations. Consequently, we limit our claims and say that the application of these methods to high frequency multivariate physiological data provides an excellent way to discover changes in physiology associated with varying physiologic state and we recommend further studies to delve into the specific ways in which this information can be put to practical use. These studies could involve more patients given well-defined doses of individual pressors and would enable the use of these methods to deeply understand their effects. As additional comprehensive data sets become available - and indeed they are currently being generated - these methods can easily be applied in a variety of ways to provide insight into the rapidly changing physiology of ICU patients. Ideally, future studies could also produce concrete treatment recommendations based on individual patients' physiologic state. At a minimum, though, this sort of analysis could provide an additional source of data for physicians to incorporate into their normal diagnostic procedure. Furthermore, because our methods are computationally simple they lend themselves well to eventual implementation in clinical decision support systems and improved patient monitors that incorporate state information.

## Conclusions

We have shown that correlation networks can be built from physiological associations and that drugs modify these networks. Using correlation network analysis we determined that the strength and directionality of associations between clinical physiological variables changed when pressors were given, both in aggregate and in individual patients. In fact, the majority of these associations changed when pressors were given, reinforcing the notion that complex physiology cannot easily be modeled as a single set of mathematical relationships between metabolic variables. We also presented a proof of concept showing that patients have inherent differences in their physiology that could be exploited by a "personalized physiology" approach to medicine. We also demonstrated that different drugs have differential effects on physiological networks and that applying network analysis to clinical physiology provides information that could be useful in treating patients. Further studies in personalized physiology that open up the middle level of personalized medicine, a level between genes and demographics, have the potential to change the way drugs are evaluated during clinical trials and prescribed in the hospital or clinic.

## Competing interests

The authors declare they have no competing interests.

## Authors' contributions

AG and AB conceived of this study. AG performed the analysis and drafted the manuscript. MC and GM participated in data collection and interpretation of results, and conceived of the original study to generate these data. AB participated in the analysis and interpretation of the results. All authors edited and approved the final manuscript.

## Supplementary Material

Additional file 1 **Empirical cumulative distribution functions for each of the 29 variables collected in our study**. Red line indicates the distribution when pressors are being administered while the blue line indicates the time when pressors are not being administered. Click here for file

Additional file 2 **Correlation network diagram showing edges that are present and retain the same sign regardless of pressor administration**. Green edges indicate a positive Spearman correlation while red edges indicate a negative correlation. Click here for file

Additional file 3 **Correlation network diagram showing edges that are present either when pressors are being administered or not, but not in both cases**. Red/green edges indicate negative/positive correlation coefficients without pressors. Orange/blue edges indicate negative/positive correlations when pressors are being administered. Wider edges indicate stronger correlations. Click here for file

Additional file 4 **Correlation network diagram showing the edges that undergo a direction change when pressors are administered**. Green edges indicate a positive correlation when pressors are not being administered - and a negative correlation when pressors are administered. Red edges indicate the opposite. Click here for file

## Declarations

The publication costs for this article were funded by AJB using NIH R01 GM079719.

This article has been published as part of *BMC Medical Genomics *Volume 6 Supplement 2, 2013: Selected articles from the Second Annual Translational Bioinformatics Conference (TBC 2012). The full contents of the supplement are available online at http://www.biomedcentral.com/bmcmedgenomics/supplements/6/S2.
