# Supplementary figures and images for: Altering physiological networks using drugs: steps towards personalized physiology
Source: BMC Med Genomics. 2013 May 7;6(Suppl 2):S7. doi: 10.1186/1755-8794-6-S2-S7 (PMC3654899; doi:10.1186/1755-8794-6-S2-S7)

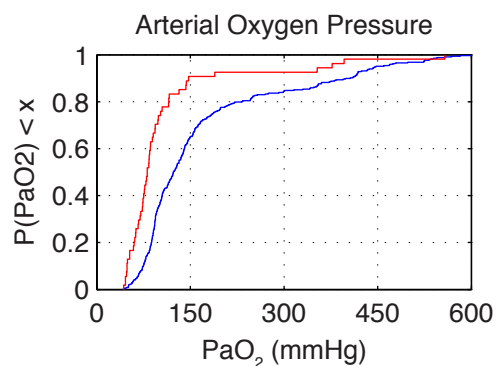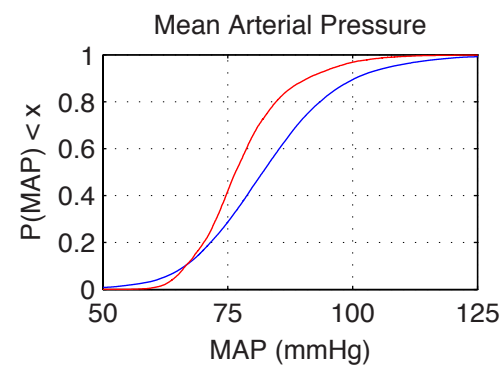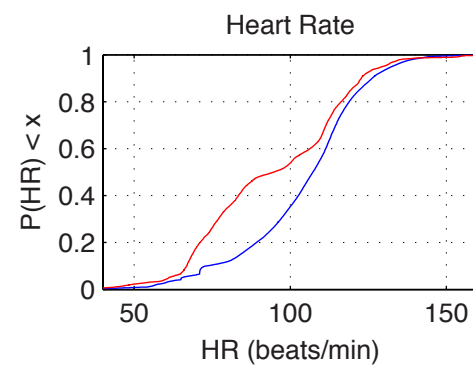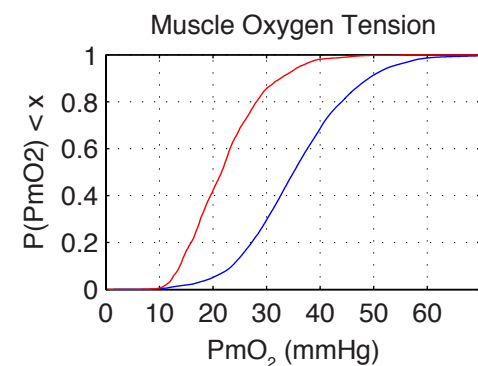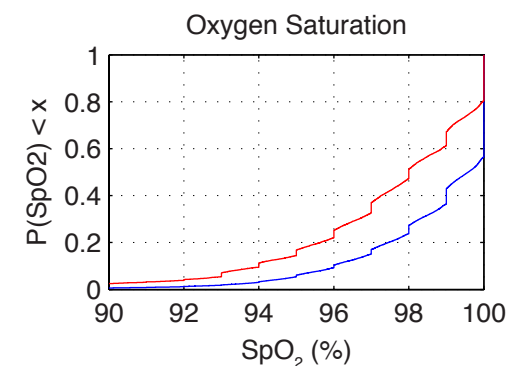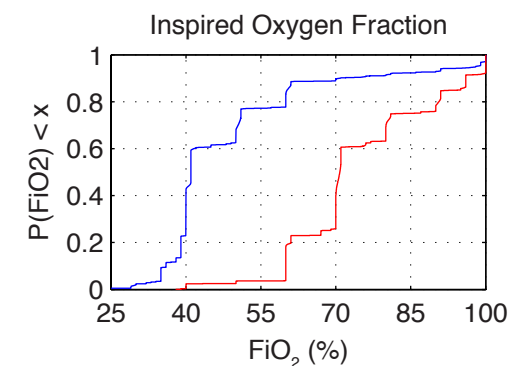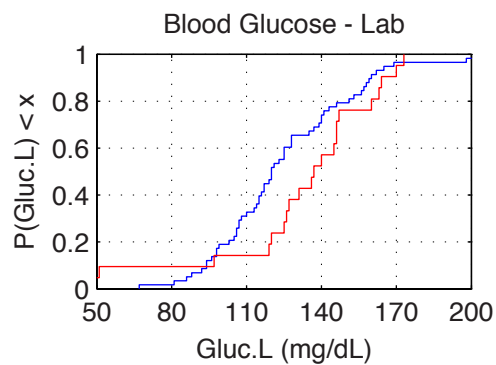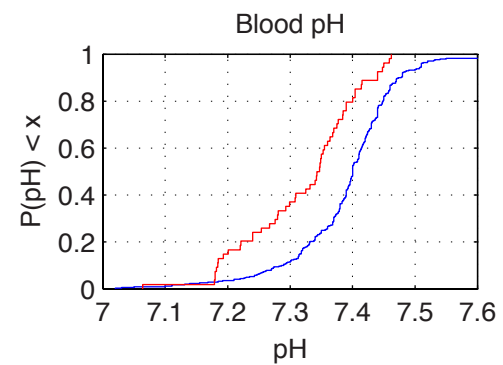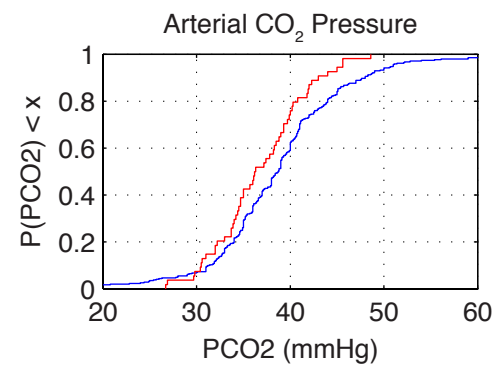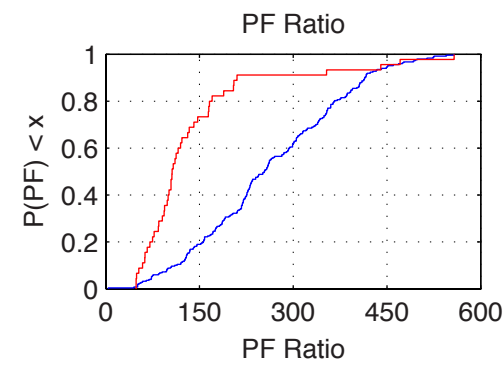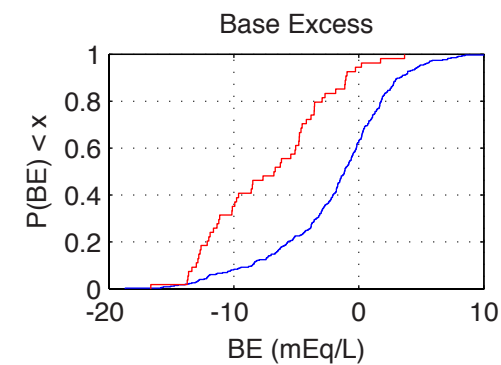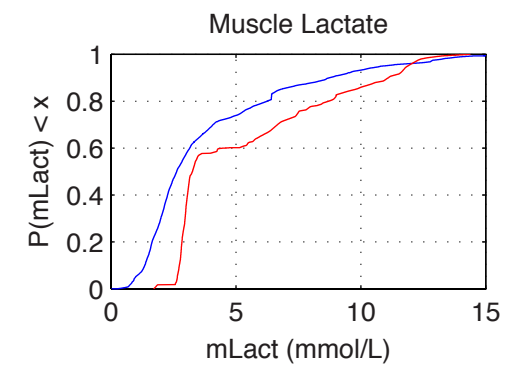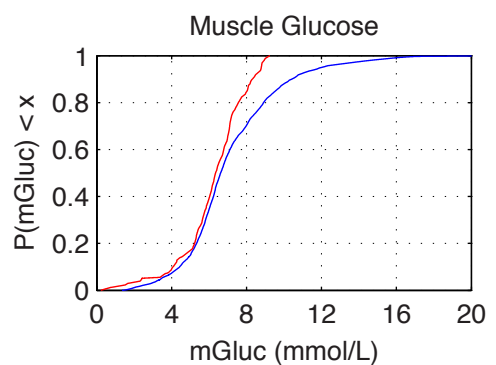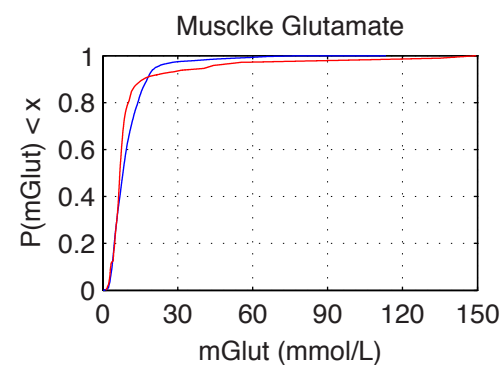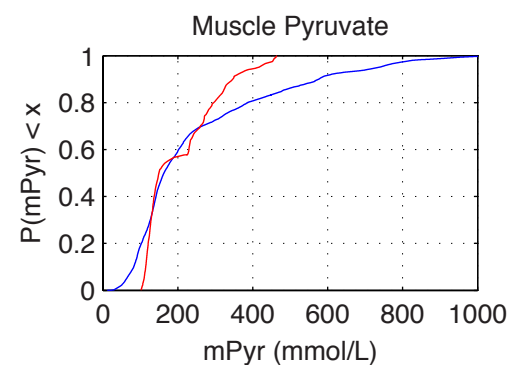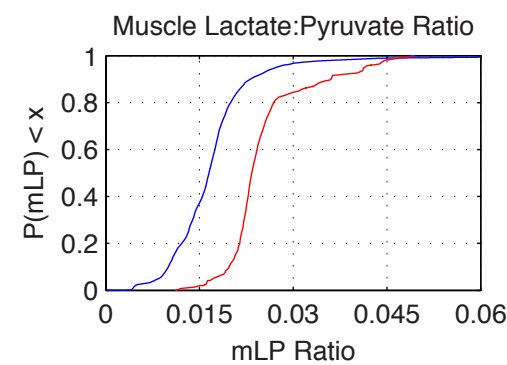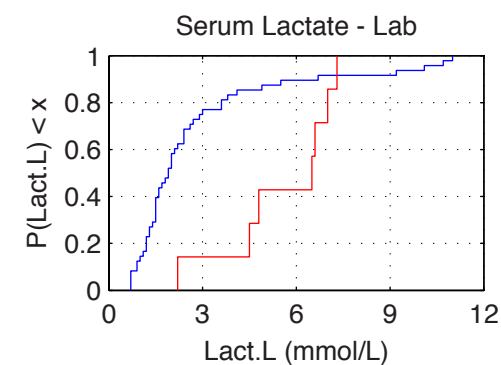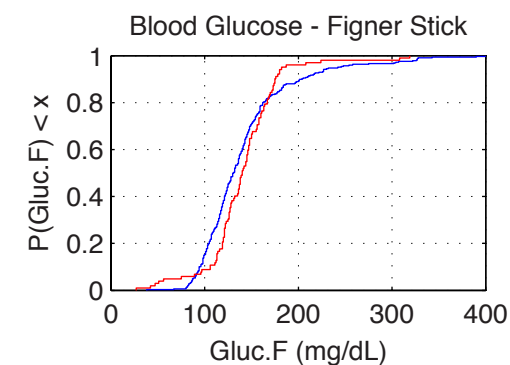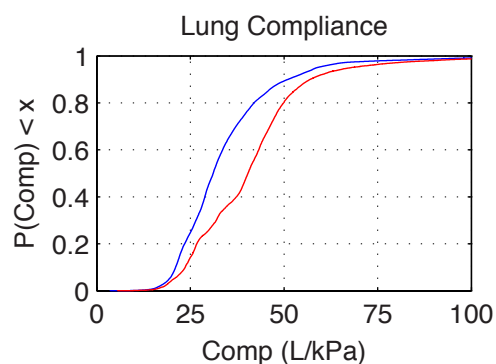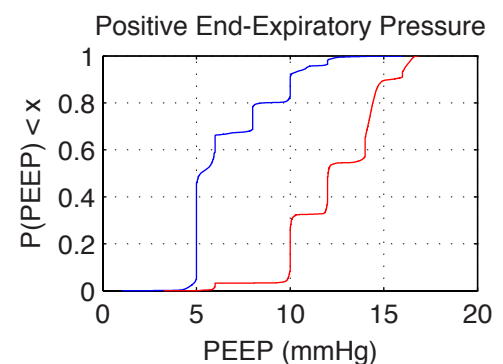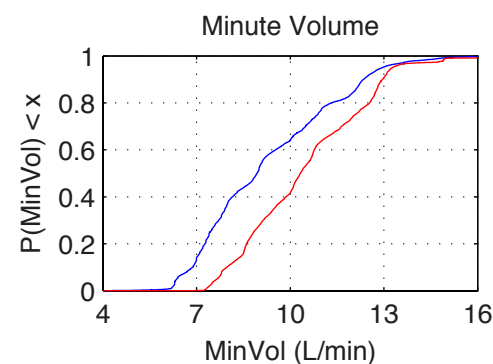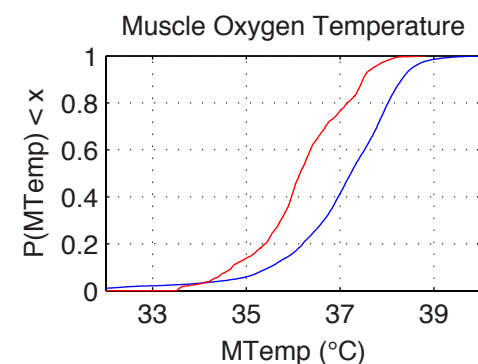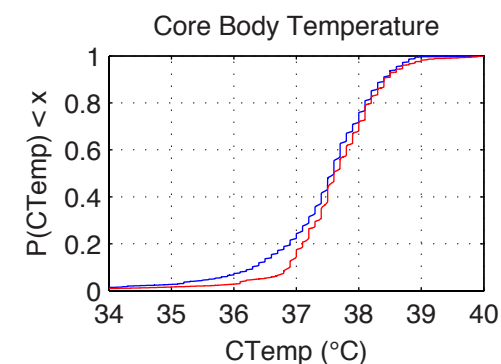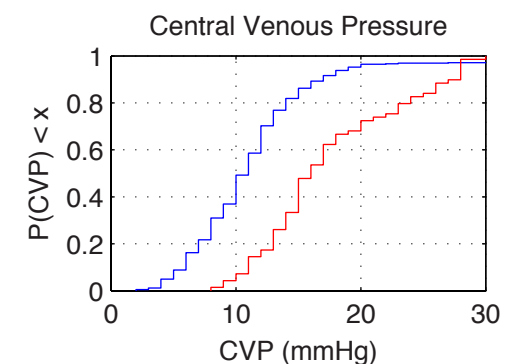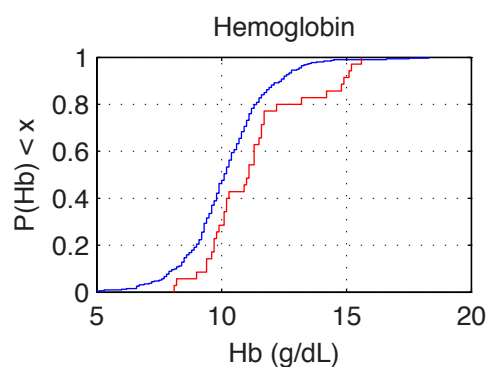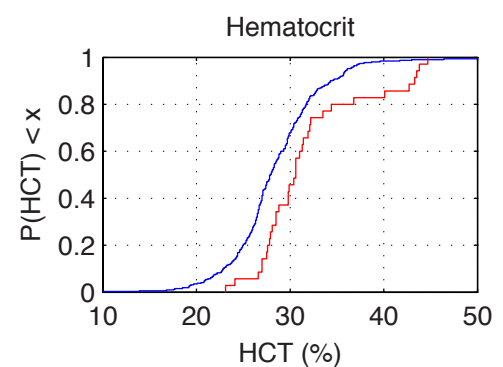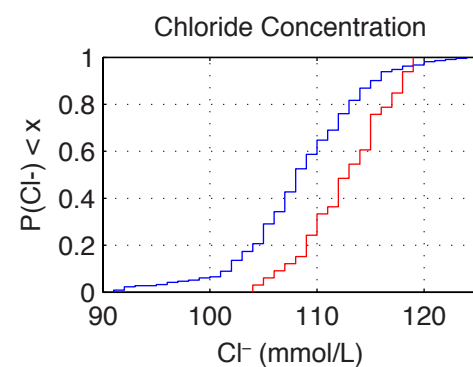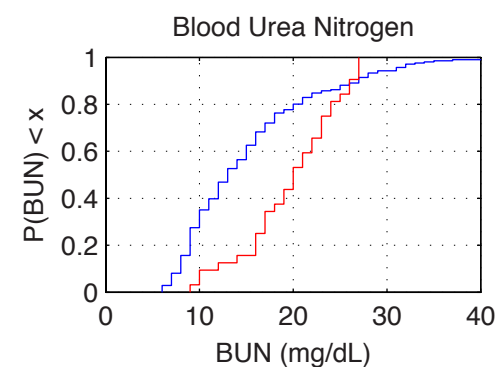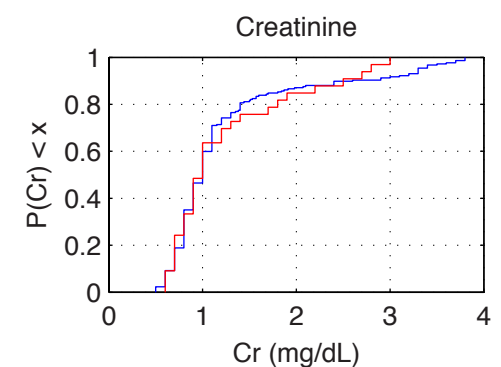

Supplement: Additional file 1 — Empirical cumulative distribution functions for each of the 29 variables collected in our study. Red line indicates the distribution when pressors are being administered while the blue line indicates the time when pressors are not being administered. [file 1755-8794-6-S2-S7-S1.pdf]

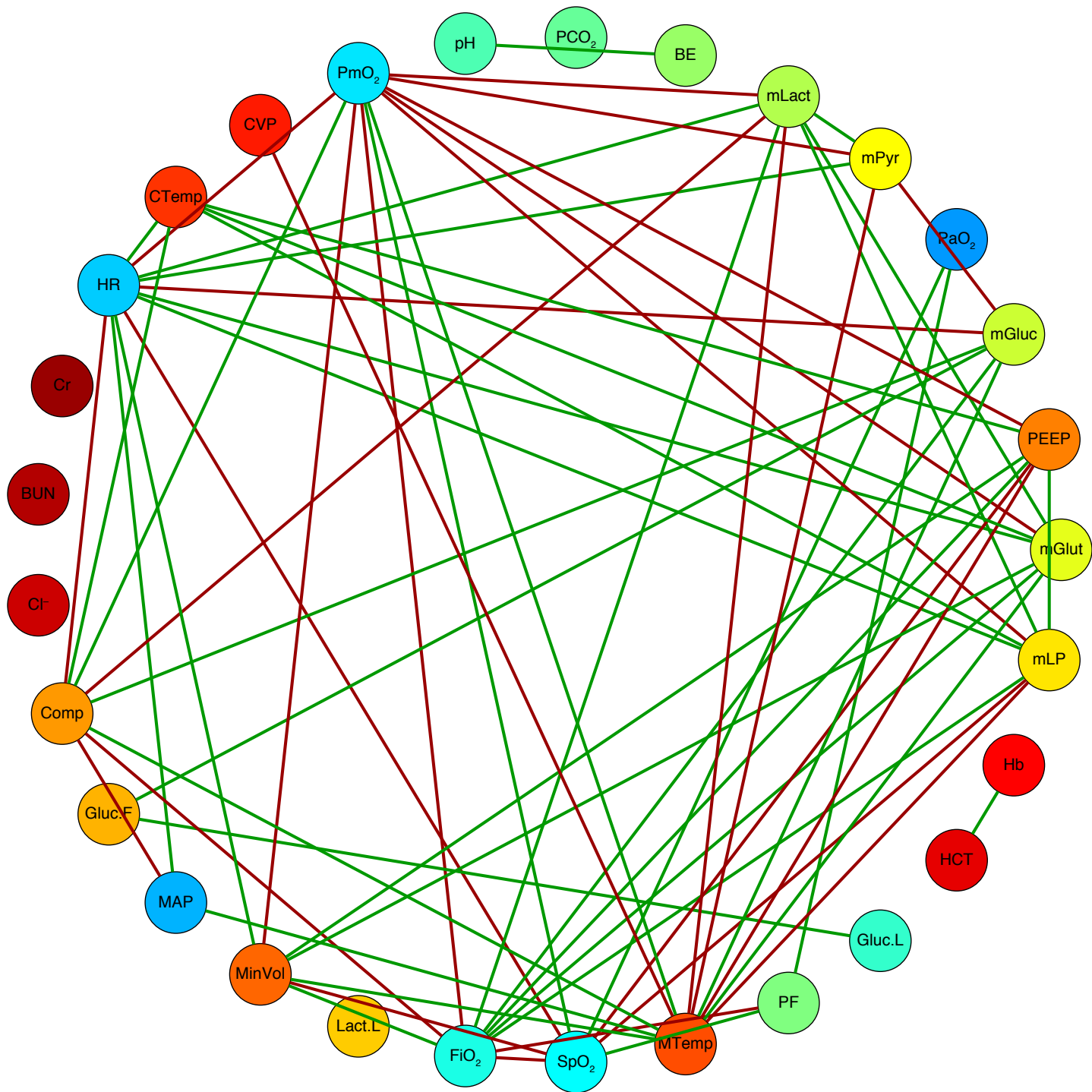

Supplement: Additional file 2 — Correlation network diagram showing edges that are present and retain the same sign regardless of pressor administration. Green edges indicate a positive Spearman correlation while red edges indicate a negative correlation. [file 1755-8794-6-S2-S7-S2.pdf]

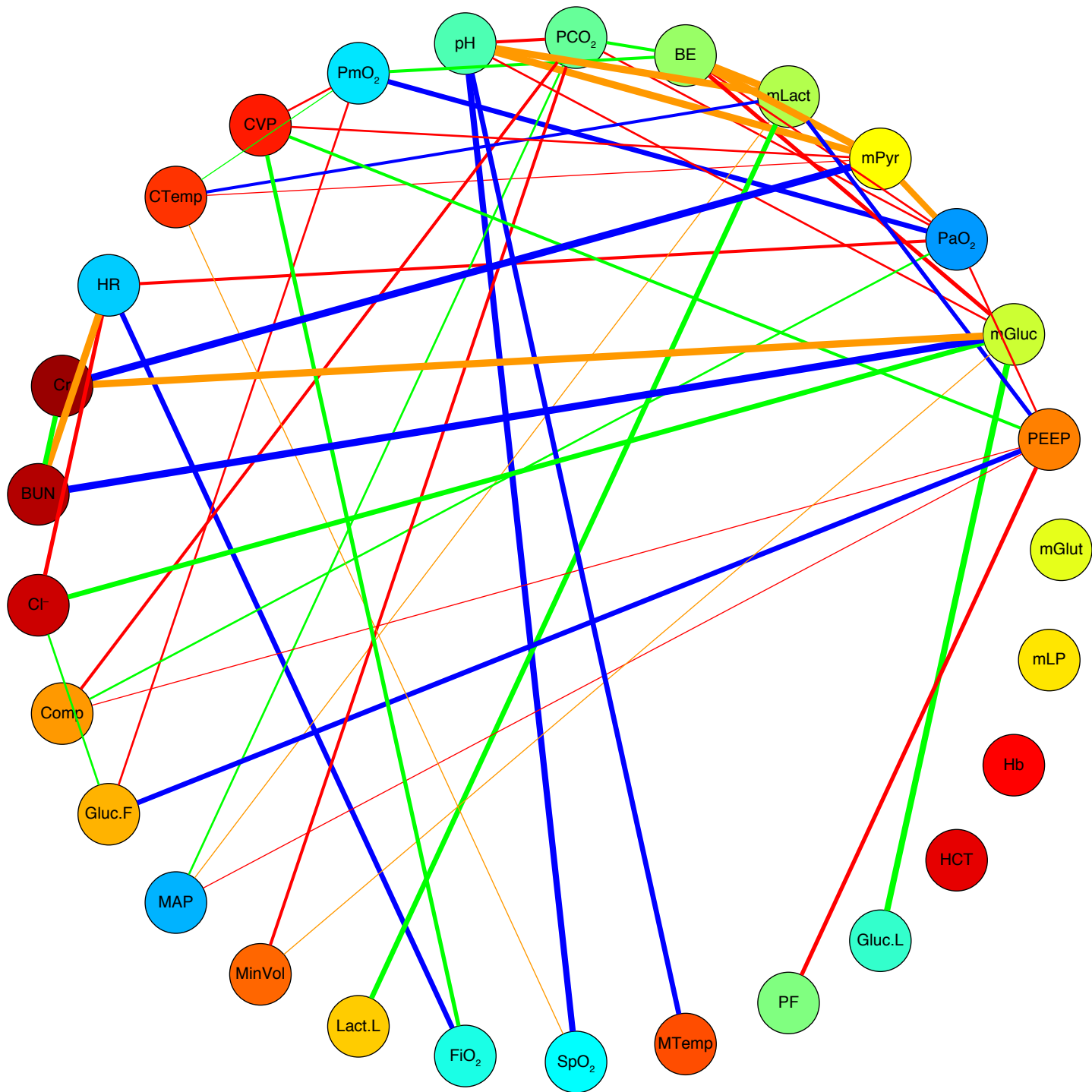

Supplement: Additional file 3 — Correlation network diagram showing edges that are present either when pressors are being administered or not, but not in both cases. Red/green edges indicate negative/positive correlation coefficients without pressors. Orange/blue edges indicate negative/positive correlations when pressors are being administered. Wider edges indicate stronger correlations. [file 1755-8794-6-S2-S7-S3.pdf]

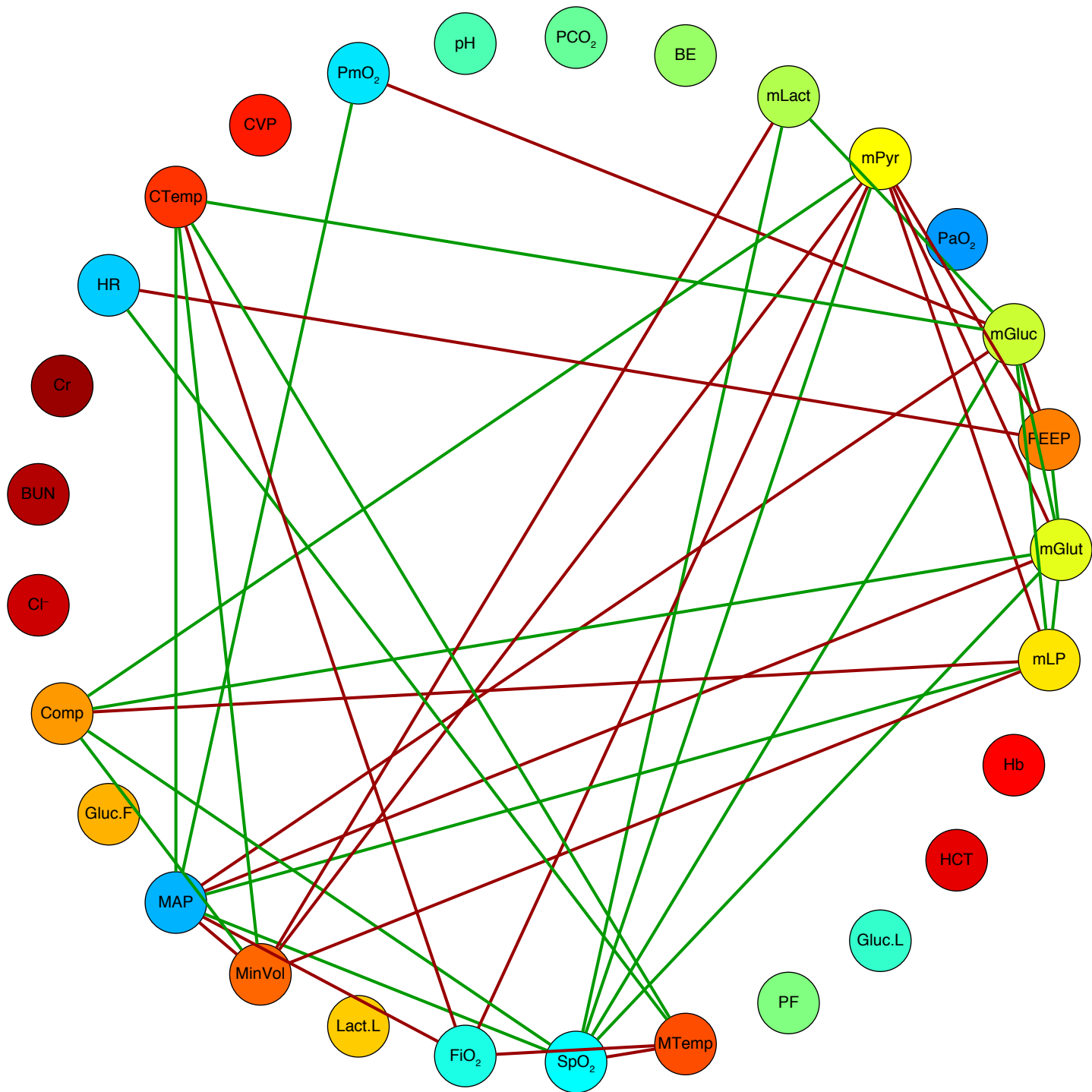

Supplement: Additional file 4 — Correlation network diagram showing the edges that undergo a direction change when pressors are administered. Green edges indicate a positive correlation when pressors are not being administered - and a negative correlation when pressors are administered. Red edges indicate the opposite. [file 1755-8794-6-S2-S7-S4.pdf]
